# Neuromechanical Assessment of Activated vs. Resting Leg Rigidity Using the Pendulum Test Is Associated With a Fall History in People With Parkinson’s Disease

**DOI:** 10.3389/fnhum.2020.602595

**Published:** 2020-12-09

**Authors:** Giovanni Martino, J. Lucas McKay, Stewart A. Factor, Lena H. Ting

**Affiliations:** ^1^W.H. Coulter Department of Biomedical Engineering, Georgia Institute of Technology, Emory University, Atlanta, GA, United States; ^2^Department of Biomedical Informatics, Emory University, Atlanta, GA, United States; ^3^Jean and Paul Amos PD and Movement Disorders Program, Department of Neurology, Emory University, Atlanta, GA, United States; ^4^Department of Rehabilitation Medicine, Division of Physical Therapy, Emory University, Atlanta, GA, United States

**Keywords:** hyperreflexia, EMG, kinematics, dual-task, activation maneuver, biomechanics, hyper-resistance, neural control

## Abstract

Leg rigidity is associated with frequent falls in people with Parkinson’s disease (PD), suggesting a potential role in functional balance and gait impairments. Changes in the neural state due to secondary tasks, e.g., activation maneuvers, can exacerbate (or “activate”) rigidity, possibly increasing the risk of falls. However, the subjective interpretation and coarse classification of the standard clinical rigidity scale has prohibited the systematic, objective assessment of resting and activated leg rigidity. The pendulum test is an objective diagnostic method that we hypothesized would be sensitive enough to characterize resting and activated leg rigidity. We recorded kinematic data and electromyographic signals from rectus femoris and biceps femoris during the pendulum test in 15 individuals with PD, spanning a range of leg rigidity severity. From the recorded data of leg swing kinematics, we measured biomechanical outcomes including first swing excursion, first extension peak, number and duration of the oscillations, resting angle, relaxation index, maximum and minimum angular velocity. We examined associations between biomechanical outcomes and clinical leg rigidity score. We evaluated the effect of increasing rigidity through activation maneuvers on biomechanical outcomes. Finally, we assessed whether either biomechanical outcomes or changes in outcomes with activation were associated with a fall history. Our results suggest that the biomechanical assessment of the pendulum test can objectively quantify parkinsonian leg rigidity. We found that the presence of high rigidity during clinical exam significantly impacted biomechanical outcomes, i.e., first extension peak, number of oscillations, relaxation index, and maximum angular velocity. No differences in the effect of activation maneuvers between groups with clinically assessed low rigidity were observed, suggesting that activated rigidity may be independent of resting rigidity and should be scored as independent variables. Moreover, we found that fall history was more common among people whose rigidity was increased with a secondary task, as measured by biomechanical outcomes. We conclude that different mechanisms contributing to resting and activated rigidity may play an important yet unexplored functional role in balance impairments. The pendulum test may contribute to a better understanding of fundamental mechanisms underlying motor symptoms in PD, evaluating the efficacy of treatments, and predicting the risk of falls.

## Introduction

Rigidity is a cardinal feature of Parkinson’s disease (PD) and its role in functional balance and gait impairment has been questioned (Wright et al., 2007; Franzén et al., 2009). Our recent work suggested that leg–but not arm, neck, or total–rigidity score is associated with frequent falls in people with PD (McKay et al., 2019). However, leg rigidity scores reflect a coarse and subjective categorization based on subitem 3.3 in the Movement Disorders Society Unified Parkinson’s Disease Rating Scale (MDS-UPDRS). Rigidity is clinically described as a constantly increased resistance to a passive or externally induced motion throughout the range of movement (Fung and Thompson, 2002). Rigidity generally responds well to dopaminergic medication and surgical interventions (Xia, 2011), and a reduction in rigidity is taken as an indicator of successful treatment. Parkinsonian patients perceive rigidity as achiness and stiffness in the muscles and joints affected, which is also used as a metric for pain and impaired mobility.

Changes in the neural state can exacerbate rigidity (Hong et al., 2007; Mendonça and Jog, 2008; Powell et al., 2011), but such effects are quantified only at the lowest range of the MDS-UPDRS. In the MDS-UPDRS, a passive movement is imposed by an examiner and the perceived stiffness is rated with an ordinal score from 0 (absent rigidity) to 4 (severe rigidity) for each arm, leg, and neck. In the “resting rigidity” condition, the subject is asked to completely relax during the assessment (Webster and Mortimer, 1977). An activation maneuver (such as finger tapping) is used in the MDS-UPDRS to evaluate “activated rigidity” only if a person exhibits no resistance when relaxed; thus activation maneuver is mainly used in only at the mildest rigidity levels (Fung et al., 2000; Powell et al., 2011). Moreover, activated rigidity has not been systematically studied in the leg, although it could play a causal role in falls (McKay et al., 2019). Thus, more sensitive and objective methods for quantifying leg rigidity are necessary to enable associations between rigidity and other biomechanical or clinical outcomes.

Here, we proposed the use of the pendulum test to objectively characterize resting vs. activated rigidity based on biomechanical outcomes and electromyographic (EMG) recordings. Various methods have been proposed in the literature to objectively quantify rigidity in PD (Eisen, 1987; Andreeva and Khutorskaya, 1996; Kirollos et al., 1996; Patrick et al., 2001; Marusiak et al., 2010; Xia et al., 2011; Powell et al., 2012; Endo et al., 2015; Zetterberg et al., 2015), but the focus has been primarily on the upper limbs. Moreover, objective metrics have not been implemented in the clinical setting because of their complexity, need for expensive devices, and time involved. In contrast, the pendulum test is a diagnostic method that allows passive joint resistance to be objectively characterized based on the pattern of lower leg movement after release from the horizontal (Wartenberg, 1951). Assessment using the pendulum test is sensitive to standard clinical measurements of spasticity in children with cerebral palsy (Fowler et al., 2000; Fee and Miller, 2004; Szopa et al., 2014; Willaert et al., 2020), multiple sclerosis patients (Bianchi et al., 1999), and stroke survivors (Brown et al., 1988; Lin and Rymer, 1991; Bohannon et al., 2009; Kristinsdottir et al., 2020). The first swing excursion is the most sensitive outcome for spasticity severity (Fowler et al., 2000; Bohannon et al., 2009; Szopa et al., 2014; Willaert et al., 2020). However, other kinematic features of the pendulum test may also provide insight. These include reductions in the number and duration of the oscillations (Fowler et al., 2000; Szopa et al., 2014), in stiffness and damping coefficients estimated by inverse kinematics (Lin and Rymer, 1991; Fee and Miller, 2004), along with abnormal bursts of activation in the quadriceps and hamstrings (Lin and Rymer, 1991; Fowler et al., 2000; Kristinsdottir et al., 2020; Willaert et al., 2020). Furthermore, the use of a computational model associated with pendulum test data is capable of dissociating the contributions of abnormal muscle tone vs. abnormal reflex excitability to spasticity (De Groote et al., 2018), revealing new insights into physiological mechanisms of spasticity. In De Groote et al. (2018) we suggested that the abnormal limb motion in children with cerebral palsy results from the interactions between muscle tone and the resulting short-range stiffness, and force-dependent reflexes. In PD, marked reductions in leg swing velocity and resting angle have been observed (Brown et al., 1988) and attributed to increased damping in simulations (Le Cavorzin et al., 2003). However, these reductions have not been associated with the degree of leg rigidity.

The pendulum test may also be sufficiently sensitive to test the level of activated rigidity which we hypothesized could potentially increase the risk of falling during activities of daily living (ADL’s). Several studies have shown that the presence of a secondary task or activation maneuver considerably enhances rigidity in people with PD (Kelly et al., 2012). The degree of the increase in parkinsonian rigidity with activation can differ from patient to patient and can be present in both on- and off- dopaminergic medication states (Fung et al., 2000; Hong et al., 2007; Shapiro et al., 2007; Powell et al., 2011). Also, different medications and dosages have been reported to have variable effects on both resting and activated rigidity (Webster and Mortimer, 1977; Kirollos et al., 1996; Relja et al., 1996; Krack et al., 2003; Shapiro et al., 2007), suggesting that different neural mechanisms could play a role in the manifestation of parkinsonian rigidity. However, the difference between activated and resting rigidity and its relationship with the degree of severity of rigidity at rest or to other clinical outcomes in PD has not been explored before.

The objective of this study was to test whether the pendulum test would be an objective and sensitive test to quantify resting and activated rigidity in PD. We hypothesized that both resting and activated rigidity in PD alter pendulum test kinematics and EMG patterns. We predicted that the biomechanical outcomes of the pendulum test, namely first swing excursion, first extension peak, number and duration of the oscillations, resting angle, relaxation index, maximum and minimum angular velocity, would be associated with leg rigidity severity in people with PD. We further predicted that an activation maneuver would alter pendulum test outcomes, but that the effects would vary from an individual to the next. Finally, as an exploratory study, we tested whether the level of activated rigidity would be associated with fall history, which would be expected if activated rigidity were a potential cause of falls.

## Materials and Methods

### Study Participants

We performed the pendulum test on 15 participants with PD. Participants were recruited from the cohort of an observational 1-year fall risk study (McKay et al., 2019). We included patients with a diagnosis of clinically defined PD who exhibited rigidity during MDS-UPDRS-III testing in the practically-defined “OFF” state (see below). Exclusion criteria were history of musculoskeletal and/or neurological disorders other than PD, inability to walk ≥3 m with or without assistance, and advanced stage dementia in which patients were unable to perform activities of daily living independently, signs of spasticity or paratonia at clinical examination. The sample size was selected to meet or exceed common recommendations of ≈10 cases/independent variable in regression analyses (Vittinghoff and McCulloch, 2007) and ≥12 cases/group in preclinical studies (Julious, 2005). PD participants were assessed in the practically defined OFF medication state, ≥12 h after their last dose of antiparkinsonian medications (Langston et al., 1992). Each participant’s neurologist signed an OFF-medication clearance form before the patient was asked to withhold their medications for this experiment. All participants provided written informed consent before participation according to protocols approved by the Institutional Review Board of Emory University.

Lower limb rigidity was evaluated at the beginning of the experimental session by a trained examiner, following the MDS-UPDRS guidelines: rigidity in the lower extremities was tested by fully extending and flexing the knee with the patient sitting (0 = Absent, 1 = Slight or detectable only when activated by mirror or other movements, 2 = Mild to moderate, 3 = Marked, but the full range of motion easily achieved, 4 = Severe, range of motion achieved with difficulty). The participants were classified as “fallers” if they reported cases of falls in the 6 months before the data collection and were classified as “non-fallers” otherwise (McKay et al., 2019).

### Pendulum Test

**Figure 1:**
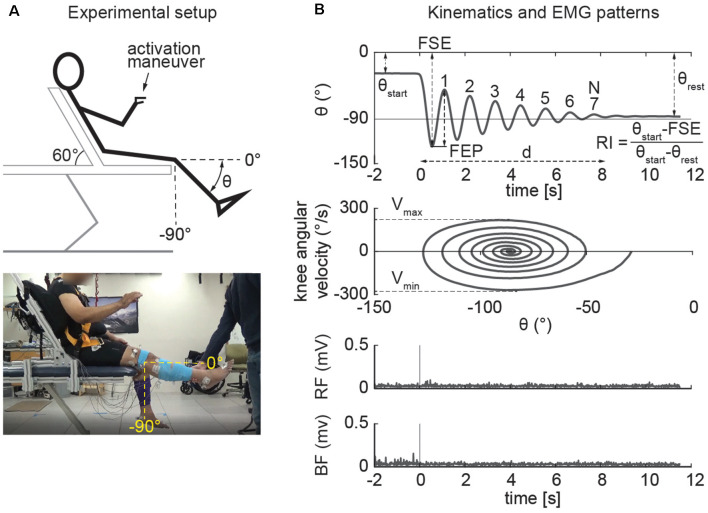
Experimental setup and outcomes of the pendulum test. The example refers to the activated condition in which the participant performs finger tapping. The pendulum test was performed with the subject sitting on a treatment table with the trunk inclined approximated 60° from the horizontal to provide a comfortable starting position **(A)**. The swinging leg behaves as a damped pendulum, oscillating several times before coming to rest. First swing excursion (FSE), number (N) and duration (d) of the oscillations, first extension peak (FEP), resting angle (θ_rest_), maximum (*V*_max_), and minimum (*V*_min_) angular velocity were assessed from kinematic data. The middle panels show the typical “whirlpool” pattern of angular velocity against angle data. EMG activity of rectus femoris (RF) and biceps femoris (BF) were also recorded in a subset of participants (bottom panel; **B**).

**Figure 2:**
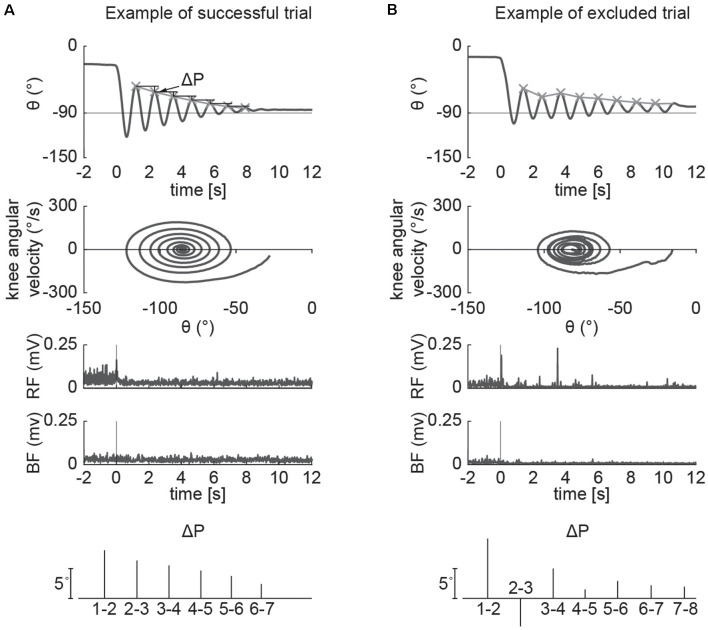
Example of successful and excluded trials. The pattern of the knee angle during the pendulum test follows an exponential decrease of the peaks, while the phase plot of angular velocity against angular displacement follows a uniform “whirlpool” shape **(A)**. The voluntary input of the participant disrupts the uniform shape of the whirlpool and causes increased variations in limb excursion peak **(B)**. We excluded the trials in which the decrement from the i-th peak to i-th+1 is not greater than the following one.

The pendulum test was performed with the subject sitting on a treatment table (Figure 1A) with the trunk inclined approximated 40° from the vertical to provide a comfortable starting position (Stillman and McMeeken, 1995). We designed a custom backrest that fits on a physical therapy table to control the posture of the participants. During the test, the examiner dropped the lower leg of the participant from the horizontal position with an extended knee joint; the lower leg was then allowed to swing freely under the influence of gravity. In each participant the pendulum test was assessed during four randomized different conditions: a baseline condition, with the subject completely relaxed and with the hands-on his/her lap, and while performing three different activation maneuvers (described below). The most rigid lower limb, as determined upon clinical examination, was assessed for each participant. Three trials were performed for each condition and a pause of 40 s was ensured between them to avoid fatigue due to the activation maneuvers. A total of 12 trials were recorded for each participant. We excluded the trials in which the participants were unable to relax, due to muscle activity that resulted in a non-monotonic exponential decrement of knee angle excursion. Specifically, using the following procedure: since the pattern of the knee angle during the pendulum test follows an exponential decrease of the peaks (Figure 2A), we excluded the trials in which the decrement from the i-th peak to i-th+1 was lower than the decrement from the i-th+1 to i-th+2 (Figure 2B). The same examiner carried out the test across all the sessions and participants.

### Activation Maneuvers

We first identified which activation maneuver was most effective in increasing rigidity during the pendulum test. We tested the effect of three different activation maneuvers (Figure 1A): finger tapping, fist-clenching, and the Jendrassik maneuver. The rationale for the incorporation of an activation maneuver lies in that activation maneuver has been shown to enhance the degree of rigidity in PD patients (Matsumoto et al., 1963; Kelly et al., 2012). The finger tapping test is one of the standard activation maneuvers used to clinically evaluate rigidity in PD (Shimoyama et al., 1990; Martínez-Martín et al., 1994) and is indicated as one of the activation maneuvers used to assess rigidity in the UPDRS scale (Fahn and Elton, 1987). The second activation maneuver consists of a sustained clenching of the fists (Meara and Cody, 1992). As an alternative to finger tapping and clenching, the Jendrassik maneuver is a common clinical test where the patient interlocks the fingers of each hand in a hook-like fashion and isometrically pulls the hands apart as strongly as possible (Ertuglu et al., 2018).

### Data Analysis

Joint kinematics were recorded using a motion capture analysis system (Vicon). Participants wore a 25-marker set according to a modified version of the Vicon’s Plug-in Gait model (Welch and Ting, 2008). Kinematic data were filtered using a second order zero-lag low pass Butterworth with a cut-off frequency of 5 Hz to remove high-frequency recording artifacts (Stillman and McMeeken, 1995; Valle et al., 2006; Lotfian et al., 2016; Ferreira et al., 2020). Knee angles were taken by measuring the absolute angle of the leg segment in the sagittal plane. Biomechanical outcomes (Figure 1B) were then calculated including first swing excursion (FSE), first extension peak (FPE), number (N) and duration (d) of the oscillations, resting angle (θ_rest_), relaxation index (RI), maximum (*V*_max_) and minimum (*V*_min_) angular velocity. The end of the oscillation was calculated by considering a cut-off of 3° toward extension (Fowler et al., 2000). These biomechanical outcomes were used in previous studies to describe the kinematic pattern of the leg during the pendulum test (Stillman and McMeeken, 1995; Fowler et al., 2000; Valle et al., 2006; Szopa et al., 2014; Lotfian et al., 2016; Whelan et al., 2018; Ferreira et al., 2020). In particular, maximum angular velocity and relaxation index (defined as the ratio between the starting angle and the resting angle of the knee) are reduced in PD (Brown et al., 1988). We also recorded EMG activity from biceps femoris (BF) and rectus femoris (RF) in a subset of participants (*n* = 10, Table 1). EMG data were collected at 1,200 Hz (Motion Lab Systems, Inc., Baton Rouge, LA, USA), high-pass filtered (35 Hz, third order zero-lag Butterworth filter) to remove motion artifact. The signal was then demeaned, rectified and low-pass filtered (40 Hz) to produce a linear envelope of the signal (Winter, 2009) as previously reported (Torres-Oviedo and Ting, 2007; Safavynia and Ting, 2013). We chose not to normalize data for within-subject interpretation of EMG activity (Powell et al., 2017).

**Table 1:** Demographic and clinical characteristics of the study participants.

ID	Age (year)	Sex	Analyzed leg side	MDS-UPDRS-III score (/132)	Leg rigidity score (/4)	Total rigidity score (/20)	Faller	Disease duration (year)	LED (mg)	EMG collected
PD01	62.5	M	R	33	1	3	N	8.5	700	N
PD02	58.3	M	L	43	3	10	N	7.3	0	N
PD03	66	F	R	23	3	7	N	2	300	N
PD04	47.4	M	L	37	4	16	Y	6.4	1,300	N
PD05	70	F	R	8	2	4	N	3.1	100	N
PD06	76	M	L	32	2	7	N	5	300	Y
PD07	64.2	M	L	33	3	6	Y	6.2	620	Y
PD08	70	M	R	52	2	7	Y	16	1,400	Y
PD09	71.1	M	L	20	1	3	N	1.1	532	Y
PD10	69.4	F	L	9	1	4	N	1.9	400	Y
PD11	55.4	M	L	28	2	6	Y	5.4	1,900	Y
PD12	81.2	M	L	38	3	7	Y	7.2	550	Y
PD13	51.4	M	R	65	2	10	Y	4.4	998	Y
PD14	72.3	F	R	28	2	5	N	5.3	700	Y
PD15	79.7	M	R	29	2	8	Y	1.7	300	Y
Mean (SD)	66.3 (9)			31.6 (15)	2.2 (0.8)	6.9 (3.2)		5.4 (3.6)	673 (506)	

### Statistical Analysis

Participants were characterized in two groups as either low rigidity (leg rigidity score from 1 to 2) or high rigidity (leg rigidity score from 3 to 4). Differences in the central tendency of clinical and demographic variables between the low and high rigidity groups and between non-fallers and fallers were assessed with *t*-tests and Chi-squared tests as appropriate. Between-groups differences in averaged outcome measures taken during rest (FSE, FPE, N, d, θ_rest_, RI, *V*_max_, and *V*_min_) were assessed with independent samples *t*-tests. Within-subject differences in averaged outcome measures (FSE, FPE, N, d, θ_rest_, RI, *V*_max_, and *V*_min_) between the resting and activated states (i.e., changes from rest to activated) were assessed with paired-samples *t*-tests. Between-groups differences in the amount of change in each outcome measure between the resting and activated states were assessed with independent samples t-tests. Cohen’s *d* parameters (Cohen, 1992) were used to evaluate the effect size on outcome measures (Table 4). Due to the exploratory nature of the study, no corrections for multiple comparisons were used .

**Table 2:** Demographic and clinical characteristics of the study participants, overall and stratified on rigidity status.

	Low rigidity (*N* = 10)	High rigidity (*N* = 5)	Entire sample (*N* = 15)	*p*-value
Age (years)				0.442
Mean (SD)	68 (9)	63 (12)	66 (10)	
Range	51–80	47–81	47–81	
Sex				0.68
F	3 (30.0%)	1 (20.0%)	4 (26.7%)	
M	7 (70.0%)	4 (80.0%)	11 (73.3%)	
MDS-UPDRS-III				0.605
Mean (SD)	30 (17)	35 (7)	32 (15)	
Range	8–65	23–43	8–65	
Total rigidity^†^				0.189
Mean (SD)	4.0 (2.0)	6.0 (3.7)	4.7 (2.7)	
Range	2–8	3–12	2–12	
Faller				0.464
N	6 (60.0%)	2 (40.0%)	8 (53.3%)	
Y	4 (40.0%)	3 (60.0%)	7 (46.7%)	
PD Duration (years)				0.787
Mean (SD)	5.2 (4.8)	5.8 (2.2)	5.4 (3.7)	
Range	1.1–16.0	2.0–7.3	1.1–16.0	
LED (mg)				0.553
Mean (SD)	733 (558)	554 (482)	673 (524)	
Range	100–1,900	0–1,300	0–1,900	

^†^*Total rigidity omits leg rigidity score*.

**Table 3:** Demographic and clinical characteristics of the study participants stratified on the prevalence of previous falls.

	Non-faller (*N* = 8)	Faller (*N* = 7)	*p*-value
Age (years)			0.454
Mean (SD)	68 (6)	64 (13)	
Range	58–76	47–81	
Sex			0.029
F	4 (50.0%)	0 (0.0%)	
M	4 (50.0%)	7 (100.0%)	
MDS-UPDRS-III			0.033
Mean (SD)	24.5 (12.1)	40.3 (13.5)	
Range	8–43	28–65	
Leg Rigidity			0.122
Mean (SD)	1.9 (0.8)	2.6 (0.8)	
Range	1–3	2–4	
Total Rigidity^†^			0.073
Mean (SD)	3.5 (1.8)	6.0 (3.1)	
Range	2–7	3–12	
Duration (years)			0.207
Mean (SD)	4.3 (2.7)	6.8 (4.5)	
Range	1.1–8.5	1.7–16.0	
LED (mg)			0.013
Mean (SD)	379 (257)	1,009 (562)	
Range	0–700	300–1,900	

*^†^Total rigidity omits leg rigidity score*.

**Table 4:** Effect size using Cohen’s *d* (Cohen, 1992).

	FSE	FEP	N	D	RI	θ_rest_	*V*_max_	*V*_min_
Rest: high vs. low rigidity	−1.25	1.31	1.27	1.11	−0.87	1.68	1.57	−0.41
Δ Rest-activated: entire sample	−0.85	0.22	0.20	−0.10	−0.41	−0.27	2.13	1.49
Δ Rest-activated: high vs. low rigidity	−0.17	−0.49	−0.19	0.39	−0.04	−0.29	−2.42	−0.60
Δ Rest-activated: fallers vs. non-fallers	0.46	−1.14	−1.41	1.23	1.35	1.25	−0.83	−0.56

## Results

Fifteen participants with PD (11 males and four females, mean age 67 ± 10 years) enrolled in the study. Demographic and clinical characteristics are shown in Table 1. No significant differences in clinical or demographic characteristics were observed between the low and high rigidity groups (Table 2). Consistent with the previous report (McKay et al., 2018), some significant differences were observed between fallers and non-fallers on Sex, Total MDS-UPDRS-III score, rigidity score, and daily levodopa equivalent dose (LED, Table 3). We excluded from the analysis of all the trials in which the participants were unable to relax the leg during the test (Figure 2). Three subjects were unable to relax during the whole session and were excluded from further analysis. The number (mean ± SD) of successful trials among participants was 2 ± 1 during resting state, 1 ± 1 during finger tapping, 2 ± 1 during fist clenching, and 2 ± 1 during the Jendrassik maneuver. Initial analyses (one-way ANOVA, *post-hoc* Tukey–Kramer) identified no significant differences between the effects of the three different activation maneuvers on the biomechanical outcomes (all *p* > 0.05). Therefore we aggregated the results of all of the activated conditions.

### Examples of Pendulum Test Kinematic Patterns

**Figure 3:**
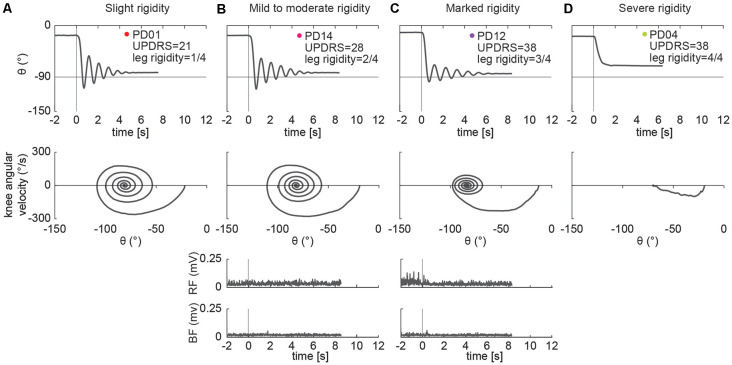
Example of pendulum test kinematic traces and EMGs in four PD individuals with increasing levels of lower leg rigidity (as measured by following the UPDRS guidelines). Slight rigidity **(A)**. Mild to moderate rigidity **(B)**. Marked rigidity **(C)**. Severe rigidity **(D)**. No EMG was recorded in PD01 and PD04.

Different kinematic patterns of the pendulum test were observed across lower leg rigidity scores. For example, in a participant with slight leg rigidity (Figure 3A, score = 1/4) the leg oscillated four times, with a first swing excursion of greater than 100°, and negative peak angular speed of about −300°/s. A participant with mild to moderate rigidity (score = 2/4) exhibited a similar pattern (Figure 3B), with the leg oscillating five times before coming to rest. Although a participant with marked rigidity (Figure 3C, score = 3/4) also had about four-leg oscillations, the first swing excursion was smaller than participants with lower rigidity scores, near 90°, and negative peak angular speed of about −230°/s. In the participant with severe rigidity (Figure 3D, score = 4/4) no oscillations were observed, with the leg slowly lowering to a less vertical resting angle than other participants.

### Low vs. High Rigidity Scores

**Figure 4:**
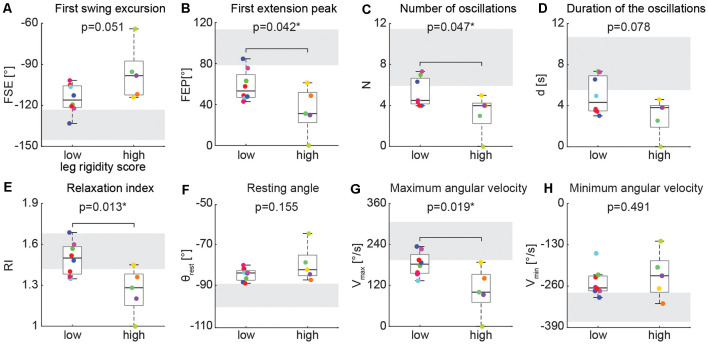
Kinematic outcomes of the pendulum test in the baseline condition. First swing excursion (FSE; **A**), first extension peak (FPE; **B**), number (N; **C**) and duration (d; **D**) of the oscillations, resting angle (θ_rest_; **E**), relaxation index (RI; **F**), maximum (*V*_max_; **G**) and minimum (*V*_max_; **H**) angular velocity. Subjects were grouped based on the rigidity score of the recorded leg: subjects with leg rigidity score from 1 to 2 (low rigidity, *n* = 8) and subjects with leg rigidity score from 3 to 4 (high rigidity, *n* = 5). Gray areas correspond to mean ± SD of biomechanical outcomes for healthy subjects estimated from Stillman and McMeeken (1995). Asterisks denote significant values (*p* < 0.05).

We found differences in the biomechanical outcomes of the pendulum test between PD participants with low leg rigidity scores (1–2) and with high leg rigidity scores (3–4) during the resting condition (Figure 4). As rigidity increased there was a significant reduction of the first extension peak (Figure 4B, 58° ± 15 vs. 34° ± 23, *p* = 0.042), several oscillations (Figure 4C, 5 ± 1 vs. 3 ± 2, *p* = 0.047), relaxation index (Figure 4E, 1.5 ± 0.1 vs. 1.3 ± 0.2, *p* = 0.013) and maximum angular velocity (Figure 4G, 182°/s ± 35 vs. 105°/s ± 69, *p* = 0.019). Although not statistically significant, the first swing excursion showed also a trend toward reduction in the high rigidity group vs. the low rigidity group (Figure 4A; −97° ± 20 vs. −115° ± 11, *p* = 0.051). Furthermore, most of the individual values for both groups fell out of the range of the mean (±SD) of the biomechanical parameters (Figure 4, gray areas) estimated from previously reported pendulum test data in healthy subjects (Stillman and McMeeken, 1995).

### Effects of Activation Maneuver

**Figure 5:**
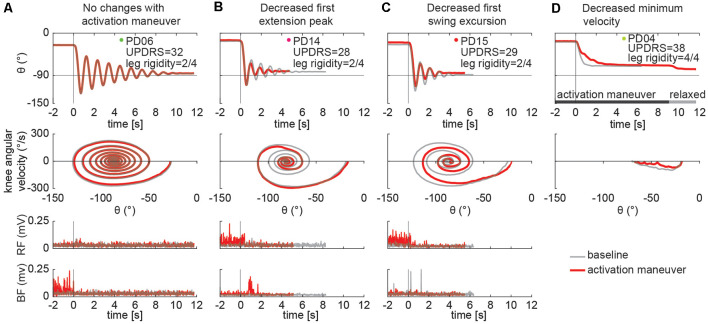
Individual specific changes in the pattern of leg movement and EMG activity among PD subjects while performing an activation maneuver (AM). In subject PD06 we found no kinematic changes with an activation maneuver **(A)**. In subject PD14 we found a decrease in the first extension peak and of the number and duration of the oscillations during AM **(B)**. In subject PD15 we found a decrease in the first swing excursion and of the number and duration of the oscillations during AM **(C)**. In the subject with severe rigidity (PD04), we found a decrease in the angular velocity of the leg during AM. No EMG recorded in PD04 **(D)**.

Individual differences in the effects of the activation maneuver were observed, even across participants with similar rigidity scores. For example, three individuals with the same rigidity score exhibited marked differences in whether and how biomechanical outcomes changed in the presence of an activation maneuver (Figures 5A–C, score = 2/2). Participant A (Figure 5A) exhibited eight oscillations of the leg during the resting condition and no changes in the kinematics during the activated condition, though increased BF tonic activity was observed before the movement. Although the other two participants with a leg rigidity score of 2 (Figures 5B–C) had a similar number of oscillations in the resting condition (*N* = 4) that was reduced during an activation maneuver (*N* = 3), they exhibited differences in other features of the pendulum test outcomes. During an activation maneuver in participant B (Figure 5B) first extension peak and maximum velocity decreased, tonic activity in both the RF and BF muscles increased, and reflexive activity in the BF was observed during the first knee extension. In Participant C (Figure 5C) a decrease in the first swing excursion, resting angle, and minimum and maximum angular velocity was observed during an activation maneuver, together with increased tonic activity in RF. Participant D had severe rigidity (Figure 5D) and did not exhibit any oscillations in either the resting or activated states, but angular velocity decreased in the presence of an activation maneuver. We also observed a change in resting angle after the end of the activation maneuver in the most severe subject (Figure 5D). We did not collect EMG data for participant D.

**Figure 6:**
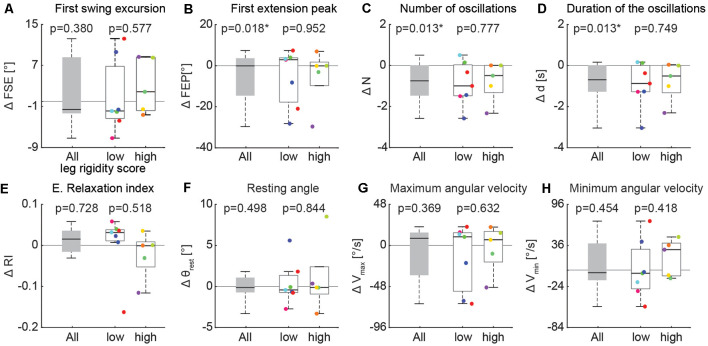
Variation of kinematic outcomes of the pendulum test during an activation maneuver. First swing excursion (FSE; **A**), first extension peak (FPE; **B**), number (N; **C**) and duration (d; **D**) of the oscillations, resting angle (θ_rest_; **E**), relaxation index (RI; **F**), maximum (*V*_max_; **G**) and minimum (*V*_min_; **H**) angular velocity. Each point represents the difference between the mean values of each outcome for one participant while performing an activation maneuver vs. the resting condition. Subjects were grouped based on the rigidity score of the recorded leg: subjects with leg rigidity score from 0 to 2 (low rigidity, *n* = 7) and subjects with leg rigidity score from 3 to 4 (high rigidity, *n* = 5). Asterisks denote significant values (*p* < 0.05).

The changes in three biomechanical outcomes in the activation vs. resting state were found to have a distribution with a mean significantly different from zero, but the magnitude of this effect did not depend on the severity of leg rigidity (Figure 6). A one-sample *t*-test revealed a significant effect of activation on the first extension peak (Figure 6B; −5.4° ± 13.4, *p* = 0.018), number of oscillations (Figure 6C; −0.8 ± 0.9, *p* = 0.013) and duration of the oscillations (Figure 6D; −0.9s ± 1.0, *p* = 0.013). A two-sample *t-test* did not reveal any significant difference in the effect of activation maneuver on the biomechanical outcomes of the pendulum test when comparing the group with low rigidity (1–2) to the group with high rigidity (3–4; Figure 6, all *p* > 0.05).

### Fallers vs. Non Fallers

**Figure 7:**
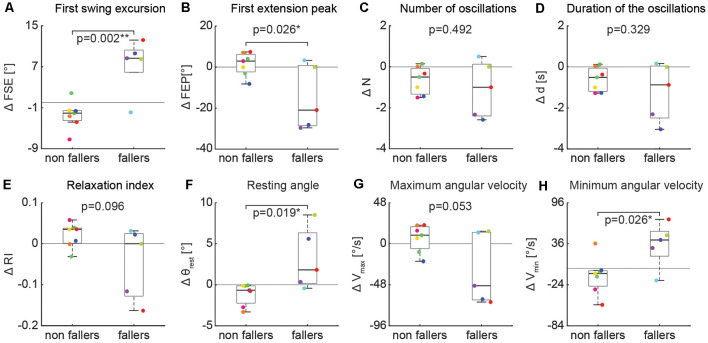
Variation of kinematic outcomes of the pendulum test during an activation maneuver in non-fallers (*n* = 7) and fallers (*n* = 5). First swing excursion (FSE; **A**), first extension peak (FPE; **B**), number (N; **C**) and duration (d; **D**) of the oscillations, resting angle (θ_rest_; **E**), relaxation index (RI; **F**), maximum (*V*_max_; **G**) and minimum (*V*_min_; **H**) angular velocity. Each point represents the difference between the mean values of each outcome for a participant while performing an activation maneuver vs. during the baseline condition. Asterisks denote significant values (*p* < 0.05).

In contrast, the effect of an activation maneuver on the biomechanical outcomes of the pendulum test was significantly different in non-fallers vs. fallers (Figure 7). In fallers compared to non-fallers, two-sample *t*-test revealed a significant decrease of first swing excursion (Figure 7A, 6.5° ± 5.3 vs. −3.1° ± 2.1, *p* = 0.002), first extension peak (Figure 7B, −13.1° ± 14.9 vs. 2.1° ± 5.7, *p* = 0.026), resting angle (Figure 7F, 2.6° ± 3.6 vs. −1.2° ± 1.4, *p* = 0.019) and minimum angular velocity (Figure 7H, 27.0°/s ± 35.1 vs. −10.4°/s ± 30.1, *p* = 0.026).

### Non-parametric Tests Performed *Post hoc*

In addition to the statistical tests described above, we performed additional non-parametric tests *post hoc* to verify that the primary results were insensitive to departures from normality. We tested the distributions of each variable entered into analyses (eight outcomes in resting and eight change scores between resting and activated for 16 total) with Shapiro–Wilk tests. Of these, five Shapiro–Wilk tests were indicative of non-normality. For these, we repeated the analyses using Wilcoxon rank-sum tests. Overall, the results were similar, with only the first extension peak test being no longer statistically significant (*p* = 0.106) when performed non-parametrically.

## Discussion

Our results demonstrate that the pendulum test is an objective measure to assess both resting and activated lower leg rigidity in people with PD. Five biomechanical metrics (first swing excursion, first extension peak, number of oscillations, relaxation index, and maximum angular velocity) describing the oscillating pattern of the leg during the pendulum test were lower in those with higher leg rigidity scores, suggesting that a simple kinematic analysis of the pendulum test is sufficient to assess leg rigidity in PD. Further, in the presence of an activation maneuver, the pendulum test biomechanical outcomes were altered to a different extent among participants suggesting a sensitivity of the pendulum test to changes in rigidity. However, the effects of the activation maneuver on biomechanical outcomes were independent of the severity of leg rigidity scores at rest. On the contrary, individuals exhibiting an effect of the activation maneuver on biomechanical outcomes experience more falls in the preceding 6 months, suggesting that increased activated rigidity could be related to increased risk of falls and highlighting the need to clinically evaluate activated rigidity independently from resting rigidity. Individual differences in the changes in biomechanics and muscle activity when performing the activation maneuver also suggest that there may be diverse underlying neural mechanisms at play that warrant further investigation. We conclude that activated rigidity may play an important yet unexplored role in fall risk in people with PD. The pendulum test may provide an important objective evaluation of resting and activated rigidity that may contribute to a better understanding of fundamental mechanisms underlying motor symptoms in PD and their fluctuations, evaluate the efficacy of treatments, and potentially reduce the risk of falls.

This is the first study to demonstrate that biomechanical outcomes of the pendulum test may be useful in objectively assessing the severity of leg rigidity among PD participants. A few studies have described the abnormal pattern (i.e., reduced number of oscillations, maximum velocity and relaxation index) of the pendulum test in people with leg rigidity (Schwab, 1963; Brown et al., 1988; Le Cavorzin et al., 2003), but its relationship to the severity of rigidity has not been assessed previously. Here we found that the first extension peak, the number of oscillations, relaxation index, and maximum angular velocity were significantly decreased in PD people with marked rigidity compared to PD people with moderate rigidity. Further, we observed that our less rigid group had altered pendulum test kinematics concerning outcomes reported previously in healthy adults (Stillman and McMeeken, 1995), although some of the differences could be attributable to aging and require further exploration. In this pilot study, we focused on the differences in biomechanical outcomes between low and high rigidity groups, but larger studies will be required to assess the sensitivity, reliability, and repeatability to validate these measures for clinical assessment of rigidity and account for potential confounding factors (McKay et al., 2018).

The pendulum test has the potential to be an objective, simple, fast, practical, and affordable diagnostic method to evaluate rigidity. Expert neurologists can commit an error of up to 20% in assessing rigidity (Rizzo et al., 2016). Other instrumented clinical tests allow the evaluation of objective continuous parameters overcoming the limitations of the UPDRS rating scale (i.e., low resolution, inter-and intra- rater unreliability, ceiling effect), which include surface electromyography (Eisen, 1987; Andreeva and Khutorskaya, 1996), myometry (Marusiak et al., 2010), and/or torque measuring devices (Kirollos et al., 1996; Patrick et al., 2001; Endo et al., 2009; Xia et al., 2011; Powell et al., 2012; Zetterberg et al., 2015). However, to the best of our knowledge, all the methods previously proposed in the literature focused on the objective quantification of upper limb rigidity (Ferreira-Sánchez et al., 2020). The biomechanical outcomes of the pendulum test can be easily evaluated through simple observation of the leg swing or by using affordable devices equipped with gyroscope (Yeh et al., 2016) or simple video source (i.e., markerless motion capture, Mathis et al., 2018), making it feasible for standard clinical practice and telemedicine. For example, automated analysis of the pendulum test could be implemented into smartphones (Prince et al., 2018) whereas prior methods require expensive additional devices, data processing, and technical assistance (Ferreira-Sánchez et al., 2020).

This study supports the idea that resting and activated rigidity should be regarded as independent variables and scored separately (Fung et al., 2000). Currently, activation maneuvers are used in clinical evaluation only to detect rigidity at an early stage, or to bring rigidity into evidence if it does not manifest at rest. In this case, the UPDRS rating system assigns a score of 1, which is not dependent on the amount of rigidity elicited by the activation maneuver, and activated rigidity is not assessed if the resting rigidity is scored at a 1 or higher. Despite several studies quantifying the effect of an activation maneuver on rigidity (Fung et al., 2000; Hong et al., 2007; Powell et al., 2011), it is not clear whether the activated rigidity is greater in people with higher resting rigidity. Here, biomechanical outcomes revealed no differences in the effect of activation maneuvers between groups with clinically assessed low and high rigidity, suggesting that the effect of the activation maneuvers may be independent of rigidity severity at rest. Heterogeneity in the manifestation of activated rigidity may further provide insight into the varied mechanisms of motor impairment in people with PD. Several factors have been suggested to contribute to the rigidity (Berardelli et al., 1983; van den Noort et al., 2017) including an increase in involuntary background activation (Marsden, 1982), changes in non-neural muscle tissue properties (Dietz et al., 1981), increased stretch reflexes (Tatton and Lee, 1975; Meara and Cody, 1993; Xia et al., 2016) and presence of shortening reaction (Lee et al., 2002; Xia et al., 2009). Furthermore, asymmetrical patterns of rigidity can be present among extensors and flexors (Meara and Cody, 1993; Xia et al., 2009). Although we recorded EMG activity only in a subsample of participants, our exploratory results suggest that increased tonic and reflex activity could be not mutually exclusive manifestations of rigidity. Indeed, while some individuals showed an increase of tonic activity in either flexors or extensors during an activation maneuver, others had an increase of muscle activity time-locked to the kinematic trajectories, consistent with reflexive activity.

Clinical assessment of activated rigidity—even when rigidity at rest is present—could help identify individuals with a higher risk of falls. The recently identified relationship between leg rigidity and falls in people with PD (McKay et al., 2019) highlights the need for more objective and continuous measures of leg rigidity (Ward et al., 1983). Here, we showed that the effects of the activation maneuver on pendulum test kinematics are greater in fallers compared to non-fallers, suggesting a potential role of activated rigidity in postural instability. Activated rigidity likely reflects a more realistic scenario of daily life, in which different concurrent tasks (such as talking or carrying an object) are performed during balance control. We found no significant difference among the tested activation maneuvers, supporting previous findings of the non-specificity of activation procedures (Hong et al., 2007). Moreover, several studies have shown that treatments can have a differential efficacy in reducing resting and activated rigidity (Webster and Mortimer, 1977; Caligiuri and Galasko, 1992; Kirollos et al., 1996; Krack et al., 2003; Shapiro et al., 2007). As such, the monitoring of activated rigidity could help predict the functional motor impairments arising during daily activities that may lead to falls, although this relationship is still unknown. The efficacy of treatments and rehabilitative interventions aimed at reducing rigidity should take into account individual responsiveness to both resting and activated rigidity. The pendulum test could help identify the extent to which multiple impaired physiological mechanisms manifest from patient to patient, representing a potential approach to understand the functional implications of resting and activated rigidity on movement.

This study had several limitations. First, the small sample size did not allow us to assess the sensitivity and validity of the pendulum test as a tool to measure the severity of resting and activated rigidity in PD. Increasing the cohort would also account for possible confounding factors such as the absence of women in the fallers group, and the difference in Total MDS-UPDRS-III score, rigidity score, and LED between the fallers and non-fallers groups. Second, we did not collect detailed information about the nature of the falls when reported retrospectively, which may be unreliable. However, studies have shown that falling frequency, situation (i.e., during sitting/standing, walking and turning), severity and direction could help in interpreting the mechanisms leading to falls in PD (Hiorth et al., 2013; Youn et al., 2017). Last, we collected EMG only in a subset of participants (*n* = 10, two of which were excluded from the analysis since they were unable to relax). This limited our ability to assess the neuropathological mechanisms that are mainly responsible for the abnormal pattern of the leg during the pendulum test in PD.

In conclusion, our results suggest that the biomechanical analysis of the pendulum test may provide an objective method to assess rigidity in people with PD that could be implemented into clinical practice. We also showed that the effect of an activation maneuver on pendulum test kinematics is variable across PD participants and independent from the leg rigidity score evaluated at rest, and thus should be scored separately. The importance of assessing activated rigidity is also highlighted by the increased effects of an activation maneuver in fallers compared to non-fallers PD groups. Further studies are necessary to elucidate the neurophysiological mechanisms of rigidity causing the abnormal pattern of the pendulum test in PD.

## Data Availability Statement

The raw data supporting the conclusions of this article will be made available by the authors, without undue reservation.

## Ethics Statement

The studies involving human participants were reviewed and approved by Institutional Review Board of Emory University. The patients/participants provided their written informed consent to participate in this study. Written informed consent was obtained from the individual(s) for the publication of any potentially identifiable images or data included in this article.

## Author Contributions

LT and GM contributed to the conception of the study and wrote the original draft of the manuscript. GM, JM and LT contributed to the design of the experiments. GM performed the experiments and analyzed the data. GM and JM performed the statistical analysis. JM and SF provided clinical data. All authors contributed to the article and approved the submitted version.

## Conflict of Interest

The authors declare that the research was conducted in the absence of any commercial or financial relationships that could be construed as a potential conflict of interest.

## References

[B1] Andreeva Y. Khutorskaya O. (1996). Application EMGs spectral analysis method for the objective diagnosis of different clinical forms of Parkinson’s disease. Electromyogr. Clin. Neurophysiol. 36, 187–192. 8737941

[B2] Berardelli A. Sabra A. F. Hallett M. (1983). Physiological mechanisms of rigidity in Parkinson’s disease. J. Neurol. Neurosurg. Psychiatry 46, 45–53. 10.1136/jnnp.46.1.45 6842199 PMC1027262

[B3] Bianchi L. Monaldi F. Paolucci S. Iani C. Lacquaniti F. (1999). Quantitative analysis of the pendulum test: application to multiple sclerosis patients treated with botulinum toxin. Funct. Neurol. 14, 79–92. 10399620

[B4] Bohannon R. W. Harrison S. Kinsella-Shaw J. (2009). Reliability and validity of pendulum test measures of spasticity obtained with the Polhemus tracking system from patients with chronic stroke. J. Neuroeng. Rehabil. 6:30. 10.1186/1743-0003-6-30 19642989 PMC2724410

[B5] Brown R. A. Lawson D. A. Leslie G. C. MacArthur A. MacLennan W. J. McMurdo M. E.. (1988). Does the Wartenberg pendulum test differentiate quantitatively between spasticity and rigidity? A study in elderly stroke and Parkinsonian patients. J. Neurol. Neurosurg. Psychiatry 51, 1178–1186. 10.1136/jnnp.51.9.1178 3225601 PMC1033023

[B6] Caligiuri M. P. Galasko D. R. (1992). Quantifying drug-induced changes in parkinsonian rigidity using an instrumental measure of activated stiffness. Clin. Neuropharmacol. 15, 1–12. 10.1097/00002826-199202000-00001 1576594

[B7] Cohen J. (1992). A power primer. Psychol. Bull. 112, 155–159. 10.1037/0033-2909.112.1.155 19565683

[B8] De Groote F. Blum K. P. Horslen B. C. Ting L. H. (2018). Interaction between muscle tone, short-range stiffness and increased sensory feedback gains explains key kinematic features of the pendulum test in spastic cerebral palsy: a simulation study. PLoS One 13:e0205763. 10.1371/journal.pone.0205763 30335860 PMC6193683

[B9] Dietz V. Quintern J. Berger W. (1981). Electrophysiological studies of gait in spasticity and rigidity. Evidence that altered mechanical properties of muscle contribute to hypertonia. Brain 104, 431–449. 10.1093/brain/104.3.431 7272709

[B10] Eisen A. (1987). Electromyography in disorders of muscle tone. Can. J. Neurol. Sci. 14, 501–505. 10.1017/s0317167100037999 3315152

[B11] Endo T. Okuno R. Yokoe M. Akazawa K. Sakoda S. (2009). A novel method for systematic analysis of rigidity in Parkinson’s disease. Mov. Disord. 24, 2218–2224. 10.1002/mds.22752 19768729

[B12] Endo T. Yoshikawa N. Fujimura H. Sakoda S. (2015). Parkinsonian rigidity depends on the velocity of passive joint movement. Parkinsons Dis. 2015:961790. 10.1155/2015/961790 26788403 PMC4695671

[B13] Ertuglu L. A. Karacan I. Yilmaz G. Türker K. S. (2018). Standardization of the Jendrassik maneuver in Achilles tendon tap reflex. Clin. Neurophysiol. Pract. 3, 1–5. 10.1016/j.cnp.2017.10.003 30214998 PMC6133913

[B14] Fahn S. Elton R. L. (1987). “UPDRS program members. Unified Parkinson’s disease rating scale,” in Recent Developments in Parkinson’s Disease, ed. Fahn S. (Florham, NJ: Macmillian Healthcare), 153–163.

[B15] Fee J. W. Jr. Miller F. (2004). The Leg Drop Pendulum Test performed under general anesthesia in spastic cerebral palsy. Dev. Med. Child Neurol. 46, 273–281. 10.1111/j.1469-8749.2004.tb00482.x 15077705

[B16] Ferreira D. M. Liang H. Wu J. (2020). Knee joint kinematics of the pendulum test in children with and without Down syndrome. Gait Posture 76, 311–317. 10.1016/j.gaitpost.2019.12.025 31887704

[B17] Ferreira-Sánchez M. D. R. Moreno-Verdú M. Cano-de-la-Cuerda R. (2020). Quantitative measurement of rigidity in Parkinson’s disease: a systematic review. Sensors 20:880. 10.3390/s20030880 32041374 PMC7038663

[B18] Fowler E. G. Nwigwe A. I. Ho T. W. (2000). Sensitivity of the pendulum test for assessing spasticity in persons with cerebral palsy. Dev. Med. Child Neurol. 42, 182–189. 10.1017/s0012162200000323 10755458

[B19] Franzén E. Paquette C. Gurfinkel V. S. Cordo P. J. Nutt J. G. Horak F. B. (2009). Reduced performance in balance, walking and turning tasks is associated with increased neck tone in Parkinson’s disease. Exp. Neurol. 219, 430–438. 10.1016/j.expneurol.2009.06.013 19573528 PMC2775914

[B20] Fung V. S. Burne J. A. Morris J. G. (2000). Objective quantification of resting and activated parkinsonian rigidity: a comparison of angular impulse and work scores. Mov. Disord. 15, 48–55. 10.1002/1531-8257(200001)15:1<48::aid-mds1009>3.0.co;2-e 10634241

[B21] Fung V. Thompson P. (2002). Rigidity and Spasticity. Lippincott Williams and Wilkins Available online at: https://digital.library.adelaide.edu.au/dspace/handle/2440/33112. Accessed January 15, 2019.

[B22] Hiorth Y. H. Lode K. Larsen J. P. (2013). Frequencies of falls and associated features at different stages of Parkinson’s disease. Eur. J. Neurol. 20, 160–166. 10.1111/j.1468-1331.2012.03821.x 22816560

[B23] Hong M. Perlmutter J. S. Earhart G. M. (2007). Enhancement of rigidity in Parkinson’s disease with activation. Mov. Disord. 22, 1164–1168. 10.1002/mds.21524 17443709

[B24] Julious S. A. (2005). Sample size of 12 per group rule of thumb for a pilot study. Pharm. Stat. 4, 287–291. 10.1002/pst.185

[B25] Kelly V. E. Eusterbrock A. J. Shumway-Cook A. (2012). A review of dual-task walking deficits in people with Parkinson’s disease: motor and cognitive contributions, mechanisms, and clinical implications. Parkinsons Dis. 2012:918719. 10.1155/2012/918719 22135764 PMC3205740

[B26] Kirollos C. Charlett A. O’Neill C. J. Kosik R. Mozol K. Purkiss A. G.. (1996). Objective measurement of activation of rigidity: diagnostic, pathogenetic and therapeutic implications in parkinsonism. Br. J. Clin. Pharmacol. 41, 557–564. 10.1046/j.1365-2125.1996.38313.x 8799522 PMC2042619

[B27] Krack P. Batir A. Van Blercom N. Chabardes S. Fraix V. Ardouin C.. (2003). Five-year follow-up of bilateral stimulation of the subthalamic nucleus in advanced Parkinson’s disease. N. Engl. J. Med. 349, 1925–1934. 10.1056/NEJMoa035275 14614167

[B28] Kristinsdottir K. Magnusdottir G. Chenery B. Gudmundsdottir V. Gudfinnsdottir H. K. Karason H.. (2020). Comparison of spasticity in spinal cord injury and stroke patients using reflex period in pendulum test. Eur. J. Transl. Myol. 30:8907. 10.4081/ejtm.2019.8907 32499899 PMC7254420

[B29] Langston J. W. Widner H. Goetz C. G. Brooks D. Fahn S. Freeman T.. (1992). Core assessment program for intracerebral transplantations (CAPIT). Mov. Disord. 7, 2–13. 10.1002/mds.870070103 1557062

[B30] Le Cavorzin P. Carrault G. Chagneau F. Rochcongar P. Allain H. (2003). A computer model of rigidity and related motor dysfunction in Parkinson’s disease. Mov. Disord. 18, 1257–1265. 10.1002/mds.10532 14639665

[B31] Lee H.-M. Huang Y.-Z. Chen J.-J. J. Hwang I.-S. (2002). Quantitative analysis of the velocity related pathophysiology of spasticity and rigidity in the elbow flexors. J. Neurol. Neurosurg. Psychiatry 72, 621–629. 10.1136/jnnp.72.5.621 11971049 PMC1737886

[B32] Lin D. C. Rymer W. Z. (1991). A quantitative analysis of pendular motion of the lower leg in spastic human subjects. IEEE Trans. Biomed. Eng. 38, 906–918. 10.1109/10.83611 1743739

[B33] Lotfian M. Mirbagheri M. M. Kharazi M. R. Dadashi F. Nourian R. Irani A.. (2016). “Pendulum test measure correlates with gait parameters in children with cerebral palsy,” in 38th Annual International Conference of the IEEE Engineering in Medicine and Biology Society (EMBC), (Orlando, FL), 1708–1711. 10.1109/EMBC.2016.7591045 28268656

[B34] Marsden C. D. (1982). The mysterious motor function of the basal ganglia: the Robert Wartenberg Lecture. Neurology 32, 514–539. 10.1212/wnl.32.5.514 7200209

[B35] Martínez-Martín P. Gil-Nagel A. Gracia L. M. Gómez J. B. Martínez-Sarriés J. Bermejo F. (1994). Unified Parkinson’s Disease Rating Scale characteristics and structure. The Cooperative Multicentric Group. Mov. Disord. 9, 76–83. 10.1002/mds.870090112 8139608

[B36] Marusiak J. Kisiel-Sajewicz K. Jaskólska A. Jaskólski A. (2010). Higher muscle passive stiffness in Parkinson’s disease patients than in controls measured by myotonometry. Arch. Phys. Med. Rehabil. 91, 800–802. 10.1016/j.apmr.2010.01.012 20434620

[B37] Mathis A. Mamidanna P. Cury K. M. Abe T. Murthy V. N. Mathis M. W.. (2018). DeepLabCut: markerless pose estimation of user-defined body parts with deep learning. Nat. Neurosci. 21, 1281–1289. 10.1038/s41593-018-0209-y 30127430

[B38] Matsumoto K. Rossmann F. Lin T. H. Cooper I. S. (1963). Studies on induced exacerbation of Parkinsonian rigidity. J. Neurol. Neurosurg. Psychiatry 26, 27–32. 10.1136/jnnp.26.1.27 21610912 PMC495531

[B39] McKay J. L. Hackney M. E. Factor S. A. Ting L. H. (2019). Lower limb rigidity is associated with frequent falls in Parkinson’s disease. Mov. Disord. Clin. Pract. 6, 446–451. 10.1002/mdc3.12784 31392245 PMC6660233

[B40] McKay J. L. Lang K. C. Ting L. H. Hackney M. E. (2018). Impaired set shifting is associated with previous falls in individuals with and without Parkinson’s disease. Gait Posture 62, 220–226. 10.1016/j.gaitpost.2018.02.027 29571090 PMC5960619

[B41] Meara R. J. Cody F. W. (1992). Relationship between electromyographic activity and clinically assessed rigidity studied at the wrist joint in Parkinson’s disease. Brain 115, 1167–1180. 10.1093/brain/115.4.1167 1393509

[B42] Meara R. J. Cody F. W. (1993). Stretch reflexes of individual parkinsonian patients studied during changes in clinical rigidity following medication. Electroencephalogr. Clin. Neurophysiol. 89, 261–268. 10.1016/0168-5597(93)90105-x 7688690

[B43] Mendonça D. A. Jog M. S. (2008). Tasks of attention augment rigidity in mild Parkinson disease. Can. J. Neurol. Sci. 35, 501–505. 10.1017/s0317167100009197 18973070

[B44] Patrick S. K. Denington A. A. Gauthier M. J. Gillard D. M. Prochazka A. (2001). Quantification of the UPDRS rigidity scale. IEEE Trans. Neural Syst. Rehabil. Eng., 9, 31–41. 10.1109/7333.918274 11482361

[B45] Powell D. Hanson N. Threlkeld A. J. Fang X. Xia R. (2011). Enhancement of parkinsonian rigidity with contralateral hand activation. Clin. Neurophysiol. 122, 1595–1601. 10.1016/j.clinph.2011.01.010 21330199 PMC3121893

[B46] Powell D. Muthumani A. Xia R.-P. (2017). Normalizing EMG to background muscle activation masks medication-induced reductions in reflex amplitudes in Parkinsonian rigidity. J. Nat. Sci. 3:e315. Available online at: http://www.jnsci.org/index.php?journal=nsci&page=article&op=view&path%5B%5D=315. 28680966 PMC5495020

[B47] Powell D. Threlkeld A. J. Fang X. Muthumani A. Xia R. (2012). Amplitude- and velocity-dependency of rigidity measured at the wrist in Parkinson’s disease. Clin. Neurophysiol. 123, 764–773. 10.1016/j.clinph.2011.08.004 21890404 PMC3260389

[B48] Prince J. Arora S. de Vos M. (2018). Big data in Parkinson’s disease: using smartphones to remotely detect longitudinal disease phenotypes. Physiol. Meas. 39:044005. 10.1088/1361-6579/aab512 29516871

[B49] Relja M. A. Petravic D. Kolaj M. (1996). Quantifying rigidity with a new computerized elbow device. Clin. Neuropharmacol. 19, 148–156. 10.1097/00002826-199619020-00003 8777768

[B50] Rizzo G. Copetti M. Arcuti S. Martino D. Fontana A. Logroscino G. (2016). Accuracy of clinical diagnosis of Parkinson disease: a systematic review and meta-analysis. Neurology 86, 566–576. 10.1212/WNL.0000000000002350 26764028

[B51] Safavynia S. A. Ting L. H. (2013). Long-latency muscle activity reflects continuous, delayed sensorimotor feedback of task-level and not joint-level error. J. Neurophysiol. 110, 1278–1290. 10.1152/jn.00609.2012 23803325 PMC3763153

[B52] Schwab R. S. (1963). Evaluation and correlations of the wartenberg swing tests in Parkinson’s disease. Trans. Am. Neurol. Assoc. 88, 270–274. 14272254

[B53] Shapiro M. B. Vaillancourt D. E. Sturman M. M. Metman L. V. Bakay R. A. E. Corcos D. M. (2007). Effects of STN DBS on rigidity in Parkinson’s disease. IEEE Trans. Neural Syst. Rehabil. Eng. 15, 173–181. 10.1109/TNSRE.2007.896997 17601186 PMC2365513

[B54] Shimoyama I. Ninchoji T. Uemura K. (1990). The finger-tapping test. A quantitative analysis. Arch. Neurol. 47, 681–684. 10.1001/archneur.1990.00530060095025 2346396

[B55] Stillman B. McMeeken J. (1995). A video-based version of the pendulum test: technique and normal response. Arch. Phys. Med. Rehabil. 76, 166–176. 10.1016/s0003-9993(95)80026-3 7848075

[B56] Szopa A. Domagalska-Szopa M. Kidoń Z. Syczewska M. (2014). Quadriceps femoris spasticity in children with cerebral palsy: measurement with the pendulum test and relationship with gait abnormalities. J. Neuroeng. Rehabil. 11:166. 10.1186/1743-0003-11-166 25516151 PMC4277843

[B57] Tatton W. G. Lee R. G. (1975). Evidence for abnormal long-loop reflexes in rigid Parkinsonian patients. Brain Res. 100, 671–676. 10.1016/0006-8993(75)90167-5 172196

[B58] Torres-Oviedo G. Ting L. H. (2007). Muscle synergies characterizing human postural responses. J. Neurophysiol. 98, 2144–2156. 10.1152/jn.01360.2006 17652413

[B59] Valle M. S. Casabona A. Sgarlata R. Garozzo R. Vinci M. Cioni M. (2006). The pendulum test as a tool to evaluate passive knee stiffness and viscosity of patients with rheumatoid arthritis. BMC Musculoskelet. Disord. 7:89. 10.1186/1471-2474-7-89 17134492 PMC1693559

[B60] van den Noort J. C. Bar-On L. Aertbeliën E. Bonikowski M. Braendvik S. M. Broström E. W.. (2017). European consensus on the concepts and measurement of the pathophysiological neuromuscular responses to passive muscle stretch. Eur. J. Neurol. 24:981–e38. 10.1111/ene.13322 28557247

[B61] Vittinghoff E. McCulloch C. E. (2007). Relaxing the rule of ten events per variable in logistic and Cox regression. Am. J. Epidemiol. 165, 710–718. 10.1093/aje/kwk052 17182981

[B62] Ward C. D. Sanes J. N. Dambrosia J. M. Calne D. B. (1983). Methods for evaluating treatment in Parkinson’s disease. Adv. Neurol. 37, 1–7. 6858768

[B63] Wartenberg R. (1951). Pendulousness of the legs as a diagnostic test. Neurology 1, 18–24. 10.1212/wnl.1.1.18 14785753

[B64] Webster D. D. Mortimer J. A. (1977). Failure of L-dopa to relieve activated rigidity in Parkinson’s disease. Adv. Exp. Med. Biol. 90, 297–313. 10.1007/978-1-4684-2511-6_21 930749

[B65] Welch T. D. J. Ting L. H. (2008). A feedback model reproduces muscle activity during human postural responses to support-surface translations. J. Neurophysiol. 99, 1032–1038. 10.1152/jn.01110.2007 18094102

[B66] Whelan A. Sexton A. Jones M. O’Connell C. McGibbon C. A. (2018). Predictive value of the pendulum test for assessing knee extensor spasticity. J. Neuroeng. Rehabil. 15:68. 10.1186/s12984-018-0411-x 30021641 PMC6052641

[B67] Willaert J. Desloovere K. Van Campenhout A. Ting L. H. De Groote F. (2020). Movement history influences pendulum test kinematics in children with spastic cerebral palsy. Front. Bioeng. Biotechnol. 8:920. 10.3389/fbioe.2020.00920 32850754 PMC7426371

[B68] Winter D. A. (ed.). (2009). “Kinesiological electromyography,” in Biomechanics and Motor Control of Human Movement, (Hoboken, NJ: John Wiley & Sons, Ltd.), 250–280.

[B69] Wright W. G. Gurfinkel V. S. Nutt J. Horak F. B. Cordo P. J. (2007). Axial hypertonicity in Parkinson’s disease: direct measurements of trunk and hip torque. Exp. Neurol. 208, 38–46. 10.1016/j.expneurol.2007.07.002 17692315 PMC2144734

[B70] Xia R. (2011). “Physiological and biomechanical analyses of rigidity in Parkinson’s disease,” in Etiology and Pathophysiology of Parkinson’s Disease, ed. Rana A. Q. (Rijeka, Croatia: InTech), 485–506.

[B71] Xia R. Muthumani A. Mao Z.-H. Powell D. W. (2016). Quantification of neural reflex and muscular intrinsic contributions to parkinsonian rigidity. Exp. Brain Res. 234, 3587–3595. 10.1007/s00221-016-4755-9 27534863

[B72] Xia R. Powell D. Rymer W. Z. Hanson N. Fang X. Threlkeld A. J. (2011). Differentiation between the contributions of shortening reaction and stretch-induced inhibition to rigidity in Parkinson’s disease. Exp. Brain Res. 209, 609–618. 10.1007/s00221-011-2594-2 21347660 PMC3142787

[B73] Xia R. Sun J. Threlkeld A. J. (2009). Analysis of interactive effect of stretch reflex and shortening reaction on rigidity in Parkinson’s disease. Clin. Neurophysiol. 120, 1400–1407. 10.1016/j.clinph.2009.05.001 19487158

[B74] Yeh C.-H. Hung C.-Y. Wang Y.-H. Hsu W.-T. Chang Y.-C. Yeh J.-R.. (2016). Novel application of a Wii remote to measure spasticity with the pendulum test: proof of concept. Gait Posture 43, 70–75. 10.1016/j.gaitpost.2015.10.025 26669955 PMC5158180

[B75] Youn J. Okuma Y. Hwang M. Kim D. Cho J. W. (2017). Falling direction can predict the mechanism of recurrent falls in advanced Parkinson’s disease. Sci. Rep. 7:3921. 10.1038/s41598-017-04302-7 28634343 PMC5478627

[B76] Zetterberg H. Frykberg G. E. Gäverth J. Lindberg P. G. (2015). Neural and nonneural contributions to wrist rigidity in Parkinson’s disease: an explorative study using the neuroflexor. Biomed. Res. Int. 2015:276182. 10.1155/2015/276182 25685778 PMC4320927

